# Bridging single cells to organs: Mesoscale modules as fundamental units of tissue function

**DOI:** 10.1016/j.cell.2025.10.012

**Published:** 2025-11-13

**Authors:** Yun Chen, Ronald N. Germain, Ginger L. Hunter, Rajan P. Kulkarni, Arthur D. Lander, Pedro Lowenstein, Jeremy E. Purvis, Harikesh S. Wong

**Affiliations:** 1 Department of Mechanical Engineering, Johns Hopkins University, Baltimore, MD, USA; 2 Laboratory of Immune System Biology, National Institute of Allergy and Infectious Diseases, National Institutes of Health, Bethesda, MD, USA; 3 Department of Biology, Howard University, Washington, DC, USA; 4 Department of Dermatology, Cancer Early Detection Advanced Research Center (CEDAR), and Knight Cancer Institute, Oregon Health & Science University, Portland, OR, USA; 5 Operative Care Division, VA Portland Health Care System, Portland, OR, USA; 6 Department of Developmental and Cell Biology, Center for Complex Biological Systems, University of California, Irvine, Irvine, CA, USA; 7 Department of Neurosurgery, Cell and Developmental Biology, and Biomedical Engineering, The University of Michigan, Ann Arbor, MI, USA; 8 Department of Genetics, University of North Carolina at Chapel Hill, Chapel Hill, NC, USA; 9 Ragon Institute of Mass General Brigham, MIT, and Harvard, Cambridge, MA, USA; 10 Massachusetts Institute of Technology, Department of Biology, Cambridge, MA, USA

## Abstract

Recent studies at molecular and genomic scales have enriched our understanding of life’s most fundamental building block: the cell. However, bridging the gap between single-cell phenotypes and the emergent functions of tissues and organs remains a formidable challenge. Here, we suggest that the conceptual span from cells to tissues and organs is so large as to warrant intermediate stepping stones. Drawing inspiration from “network motifs”—discrete units of cell-level function that emerge from the interactions of a handful of genes or enzymes—we argue that similarly identifiable units of tissue-level function, which we term “mesoscale modules,” emerge from coordinated “interactions” among relatively small numbers of cells and their extracellular milieu. We outline several such modules and propose that a concerted effort to study them will deepen our foundational understanding of tissue and organ functions. By developing these mesoscale insights, we anticipate a more tractable and mechanistic approach to complex human conditions rooted in tissue- and organ-scale dysregulation, including developmental defects, cancer, cardiovascular disease, immune-related disorders, infectious disease, and aging.

## INTRODUCTION

Biology operates at many scales, both spatial—from molecules and cells to organisms and ecosystems—and temporal—from the fleeting lifetimes of metabolites to the long arcs of evolutionary change. An essential goal of biological research is to explain how phenomena at one scale emerge from behaviors at lower ones. To this end, powerful tools in biochemistry^1,2^ and molecular biology,^3–6^ together with advances in ‘omics technologies^7–11^ and cutting-edge microscopy,^12–14^ have revealed how interactions at the molecular scale can generate complex behaviors at the cellular scale. These advances have raised the exciting prospect that the complex physiology of tissues and organs may one day be explained in terms of the molecular processes operating inside cells—from gene-regulatory networks and signaling pathways to metabolism and cytoskeletal mechanics. The potential impact of achieving this goal is immense given that many of today’s most pressing biomedical problems—such as cancer, immune disorders, neurodegeneration, and metabolic disease—are deeply rooted in complex tissue- and organ-level functions, not merely the pathological activities of individual cells.

Yet for many of us working in this new field of “single-cell science,” the leap from cells to tissues remains daunting. Efforts such as the Human Cell Atlas^15^ and Human BioMolecular Atlas Program (HuBMap)^16^ aim to catalog every cell type, much as earlier projects like the Human Genome Project and Encyclopedia of DNA Elements (ENCODE) sought to map every gene.^15–17^ But the current task is far more complex.^18^ Genes are relatively discrete entities with defined functions, whereas cell types are heterogeneous collections of individual cells that share some features but can behave differently depending on context.^18,19^ The possible biochemical and physical interactions between cells—unfolding across space and time—create a staggering combinatorial challenge. As a result, even with today’s computational power, massive single-cell datasets, and machine learning methods, it remains difficult to understand how tissue-level properties emerge from the interactions of individual cells.

The challenge of studying emergent behaviors in biology is not a new one. By the late 20th century, researchers recognized that predicting how cells behave could not be achieved simply by cataloging genes and molecules in isolation.^20^ This realization helped establish the field of systems biology, which emphasized that the properties of complex biological systems arise from “interactions” among components rather than from the components alone. Early efforts therefore focused on constructing “whole-cell” models that attempted to simulate many molecular interactions simultaneously.^14,21,22^ While promising, these models have proved conceptually and technically challenging to generalize across biological systems, highlighting the need for more tractable and complementary approaches.^23^

Thus, researchers began seeking a “middle path” between studying single molecules or genes in isolation and attempting to model entire systems at once. This approach led to a focus on modularity—small groups of molecular interactions that perform specific tasks in a semi-independent manner from the rest of the system.^24,25^ Biologists and physicists had long proposed that molecular biology should exhibit such modularity. In this view, molecular interactions—such as enzymatic reactions and gene expression—are organized into higher-order modules for “decision-making,” just as wires and transistors inside computers are organized into logic circuits and chips.^26^ This concept was championed by early systems biologists like Uri Alon, who emphasized the value of studying small networks of interacting gene products—typically two or three nodes—which he termed “network motifs.”^27,28^ Developmental biologists, most notably Eric Davidson, also embraced modularity. He argued that gene-regulatory networks could be divided into distinct modules with specific, well-defined roles, such as “locking down” the expression of newly activated genes.^29^

Over the past three decades, the biological literature has become filled with examples of simple network motifs accomplishing complex tasks—such as “toggle switches,” “sniffers,” “fold-change detectors,” “leaky integrators,” and “noise-induced switches.”^30,31^ What made the approaches of Alon and Davidson particularly notable, however, was their recognition that these motifs do not operate in isolation but rather serve as building blocks for more complex system behaviors.^27,28,32^ This perspective helped lay the groundwork for advances in synthetic biology, where motifs are often implemented together to engineer cellular behaviors.^32^ Together, these approaches showed that bridging the molecular and cellular scales is possible by first understanding how simple modules give rise to emergent behaviors at smaller scales and then building up from there.^32–35^

This focus on modularity and emergence in biology echoes a famous debate in physics between Philip Anderson and Steven Weinberg. Anderson argued that material properties such as rigidity, which arise from interactions among atoms, could not be fully explained by particle physics alone but instead required higher-level principles. By contrast, Weinberg emphasized reductionism, suggesting that all phenomena could ultimately be understood from fundamental laws. For many in the field of systems biology, Anderson’s perspective resonates strongly: that new properties emerge at higher levels of organization and that explaining them requires concepts beyond the sum of individual components.^36^

Here, we explore whether bridging scales from the cellular to tissue and organ level might similarly benefit from identifying and explaining intermediate “mesoscale modules.” What might such units look like? To systems biologists, “network motifs” refer to small sets of molecules or genes whose interactions are complex enough to generate useful higher-order behaviors, yet simple enough that these behaviors can be explicitly derived from the quantities, properties, and interactions of their components. We now ask whether there are analogous entities at the tissue level—small collections of cells that, through interactions with one another and their environment, generate complex yet comprehensible behaviors that can be explained with similar mechanistic clarity.

In response to this question, we offer here an initial list of possible mesoscale modules, each with descriptive monikers to aid in their conceptualization (Table 1). Some of these align closely with classical histological units, such as epithelia, while others are based on less familiar patterns of tissue organization. In several cases, the interesting behaviors exhibited by these modules arise from mechanisms reminiscent of those found in biochemical and gene-regulatory network motifs—including multistability, oscillation, and feedback control (Table 1). In other cases, however, these behaviors depend more strongly on transport processes (e.g., diffusion) and mechanics that operate in qualitatively unique ways at the tissue level compared with the molecular scale. Moreover, since all tissues are composed of both cells and extracellular matrices, the modules we discuss here often depend on cell-cell and cell-matrix interactions.

Finally, we consider what a research program aimed at elucidating new module functions might look like. Such a program would provide a new framework for interpreting complex biological data, allowing researchers to connect cellular behaviors to tissue-level functions in a more systematic way.

### SELECTED EXAMPLES OF MESO-SCALE MODULES

#### Sheets

**Figure 1.**
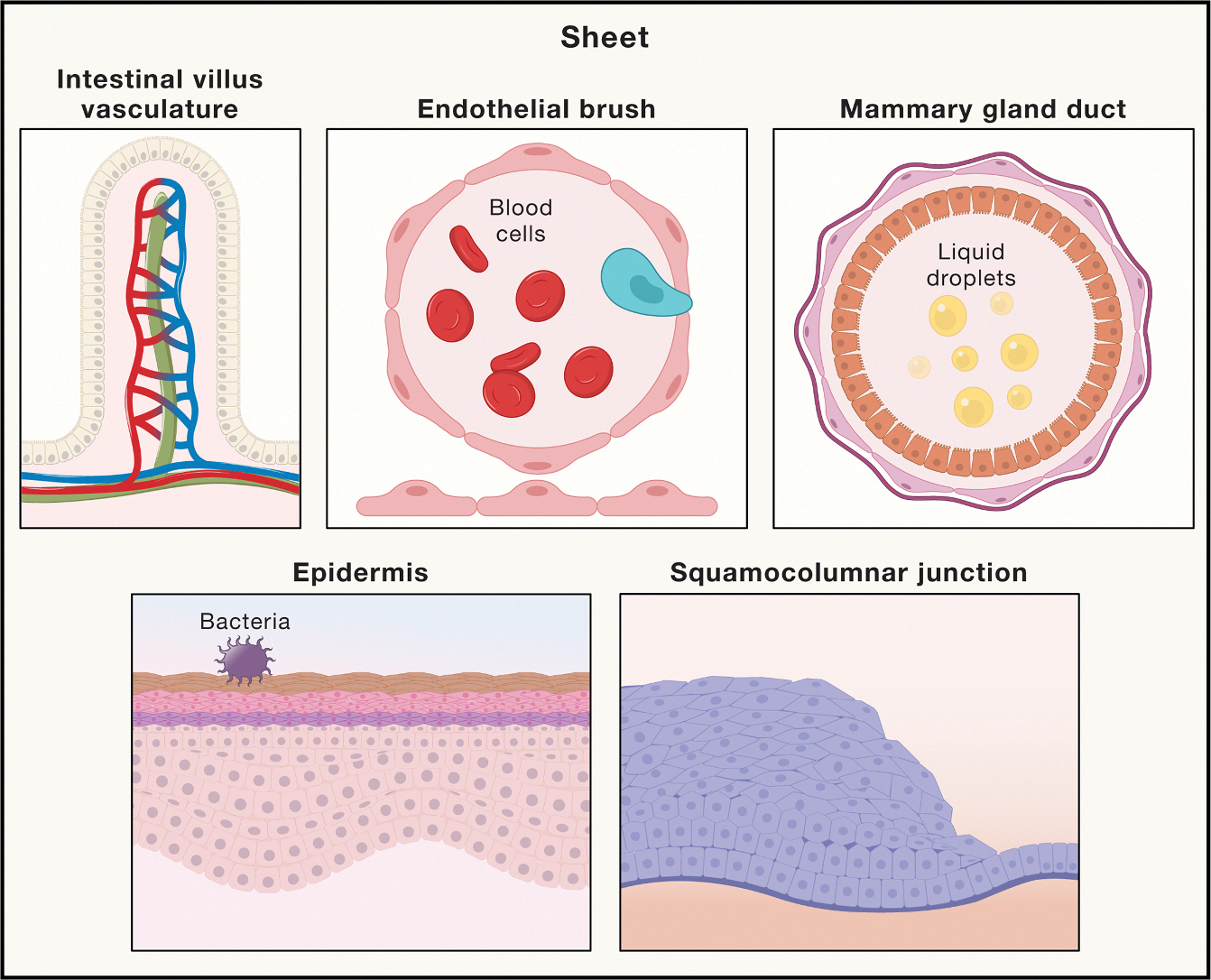
Two-dimensional arrays of cells connected by cell-cell junctions They act as selective barriers to both cell and molecular traffic. Sheets also transport a variety of substances and cells and expand the ways in which mechanical forces can be sensed and transduced into cellular behavior.

Effective tissue physiology relies on strict control of extracellular ion concentrations, osmolarity, and fluid balance. Such control is achieved in part by generating selective barriers that separate internal elements of the host from the external world, isolate solid from liquid components, and control the trafficking of molecules within and between different parts of the body. Barriers of this nature can emerge by linking multiple cells, particularly endothelial or epithelial cells, in two-dimensional arrays known as “sheets.” For example, the blood-brain barrier (BBB) is composed of tightly connected endothelial cells that prevent solutes in the blood from entering the extracellular fluid of the central nervous system.^37^ By contrast, the fenestrated endothelium of glomeruli exhibits selectively, filtering blood solutes based on size and charge while allowing water to pass freely into the kidney tubules.^38^ It should be noted that these filtering functions extend beyond the molecular scale: sheets can physically limit the entry of external pathogens into the host and control the trafficking of cells in and out of different organs (Figure 1).^39,40^

In addition to barrier and filtering properties, sheets help solve challenges related to long-range transport throughout the host. Indeed, despite being assembled as two-dimensional arrays, sheets can be folded into intricate three-dimensional (3D) anatomical structures—such as glands, blood vessels, and lymphatics—each with hollow lumens through which liquid medium flows. The net result is the ability to rapidly transport fluid, molecules, and cells over considerable distances (e.g., millimeters to meters), while largely preventing indiscriminate dispersion throughout adjacent tissue. Such large-scale trafficking is critical for a range of functions, including (1) delivery of oxygenated blood from the cardiovascular system to peripheral tissues, (2) recycling of fluid from the lymphatic system to blood, and (3) trafficking of immune cells to secondary lymphoid organs and sites of inflammation.

Sheets also endow tissues with less appreciated yet critical properties. For instance, physically linking cells in two dimensions can result in mechanical coupling, giving rise to heightened mechano-sensing capabilities that exceed that of individual cells in isolation.^41,42^ This property is essential for maintaining structural integrity when sheets are stretched or compressed by external forces or topological restrictions.^43^ Moreover, mechanical coupling improves sensitivity toward stiffness, enabling more effective durotaxis—a process in which cells migrate along an extracellular matrix (ECM) rigidity gradient.^42^ This form of migration has been suggested to be important for promoting organogenesis and wound repair.^44,45^ Lastly, sheets can facilitate lateral signal propagation and cell-cell communication throughout tissue space. Through the formation of specialized channels, interconnected cells within a sheet can rapidly transfer ions and signaling molecules between one another, enabling coordinated cell behaviors over large spatial scales.^46,47^ A striking example is observed in the epithelium of salivary glands, resulting in synchronized cycles of saliva secretion.^48^ Although experimental details are lacking, this type of signal relay is likely to operate in many biological settings, such as coordinating host defense and wound repair following a barrier breach.

The emergent functions of sheets stem largely from different junctions formed at the interface between cells. Adherens junctions, which contain mechanosensitive proteins such as vinculin, can sense curvature and force.^49,50^ Tight junctions, which contain several proteins, such as members of the claudin family, create a watertight seal between cells that controls permeability toward soluble factors across epithelium/endothelium.^51–53^ Importantly, both adherens and tight junctions are connected to actin cytoskeleton networks, which link to focal adhesions—multi-protein structures that engage with the ECM.^51^ Through these connections, cells in the sheet become mechanically coupled with each other and the environment. Finally, gap junctions are primarily composed of different connexin proteins, the combination of which forms lateral channels of varying selectivity toward ions and signaling molecules.

Although the molecular components and some of the emergent functions of sheets are well recognized, quantitative models that predict their behaviors are lacking. For instance, how do the density, composition, and spatial distribution of cell-cell junctions encode for the permeability, molecular selectivity, and mechanical coordination of sheets? Such quantitative insight could unravel the origin of pathologies involving dysregulated sheet functions, including inflammatory bowel disease, glomerulonephritis, atherosclerosis, and more. Deciphering this code should further assist in the design of therapies that can reestablish or re-engineer these functions in a wide array of disease settings.

#### Streets

**Figure 2.**
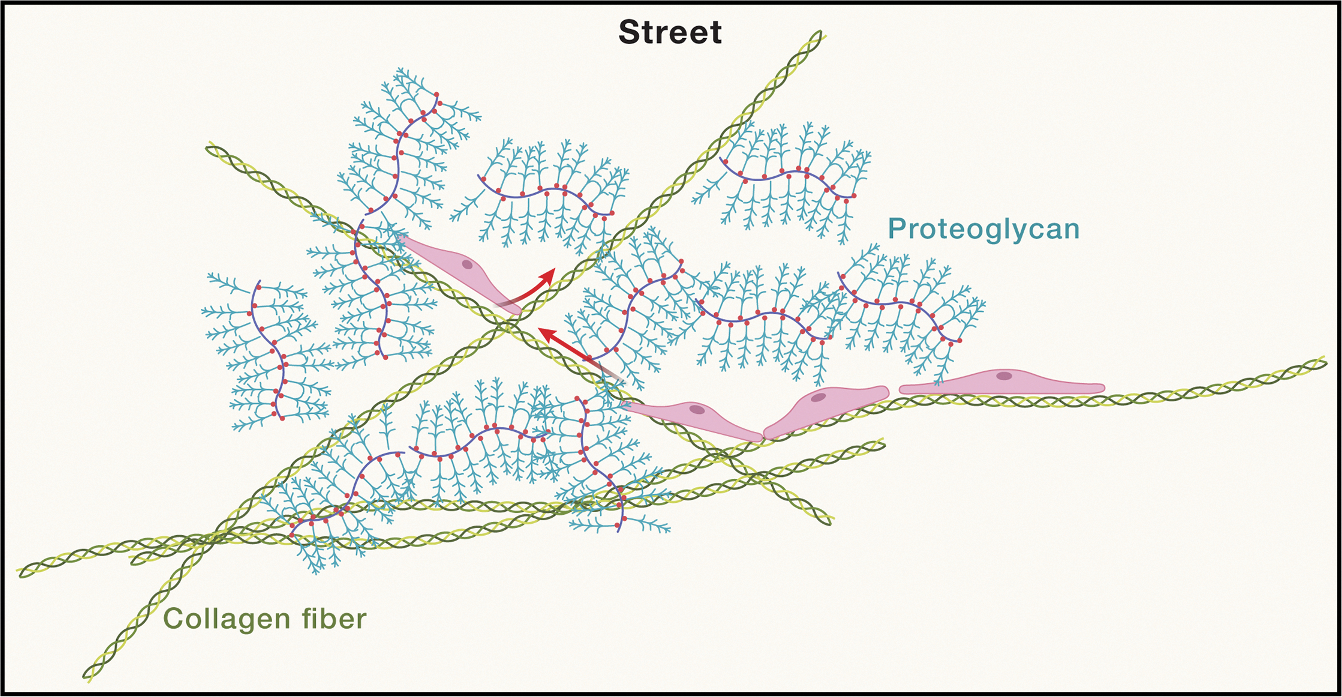
One-dimensional structures made of ECM fibrils and/or elongated, interconnected cells Streets expedite the long-range movement of cells and macromolecules.

The time it takes a random walker to cover any distance is not linear but quadratic—it increases with the square of that distance—making long-range movement inherently slow. This law of physics raises the fundamental question of how tissues enable cells to travel long distances in a short amount of time. Part of the answer lies with the acellular components of the ECM, which regulate the dimensionality of movement. In this regard, many ECM proteins—including collagens, elastins, fibronectins, and laminins—are fibrillar, providing linear paths along which cells can migrate.^54^ This behavior is exemplified by mesenchymal cells, which navigate through 3D ECM by moving on single fibers as if moving on one-dimensional avenues.^55^ These ECM-comprised “streets” reduce navigation time by providing directed paths that functionally bridge tissue space (Figure 2).

Within the complex tissue landscape, cells often need to traverse distances far greater than those afforded by ECM fibers alone. Such long-range movement can be facilitated by networks of elongated cells connected end-to-end, forming large-scale streets that streamline the dimensional paths for migrating cells. Take, for example, lymph nodes, where lymphocytes travel on networks of interconnected stromal cells in their search for antigen-bearing dendritic cells.^56^ These cellular highways constrain search to a sub-volume of the organ, markedly increasing the likelihood of lymphocytes locating their relevant target cells. Thus, streets function akin to roads of varying length scales, from short local avenues to more expansive highways, supporting travel to both immediate and distant cellular destinations.

Although streets play an important part in facilitating cellular traffic, they may also be important for the transport of smaller things, like extracellular vesicles or even soluble factors.^57^ This concept is supported by the fact that many secreted morphogens, cytokines, and chemokines can bind ECM and extracellular proteoglycans through specialized binding motifs and/or electrostatic charge.^58–60^ The diffusivity of these soluble biomolecules and extracellular vesicles^57^ is thought to be related to the ECM fiber density, pore size, orientation, and proteoglycan content.

Street functions are clearly not properties of individual cells: they most often depend on multicellular and acellular components arrayed over larger scales to reduce the degrees of freedom in space. Looking forward, it will be important to study how street parameters—such as length, angle, adhesiveness, stiffness, and porosity—have been optimized over evolutionary time to support distinct tissue properties. Understanding this optimization is crucial, as even minor street anomalies could misdirect essential cellular and molecular traffic, potentially leading to “traffic jams” or local scarcities that promote organ dysfunction. Anomalies of this nature would be particularly relevant in the context of immune surveillance or wound repair, where subtle trafficking defects could easily lead to pathogen dissemination, tumor outgrowth, or fibrosis. Understanding the robustness and fine-tuning of street parameters should also pave the way for targeted therapies that can restore proper trafficking and alleviate disease.

#### Proliferative conveyors

**Figure 3.**
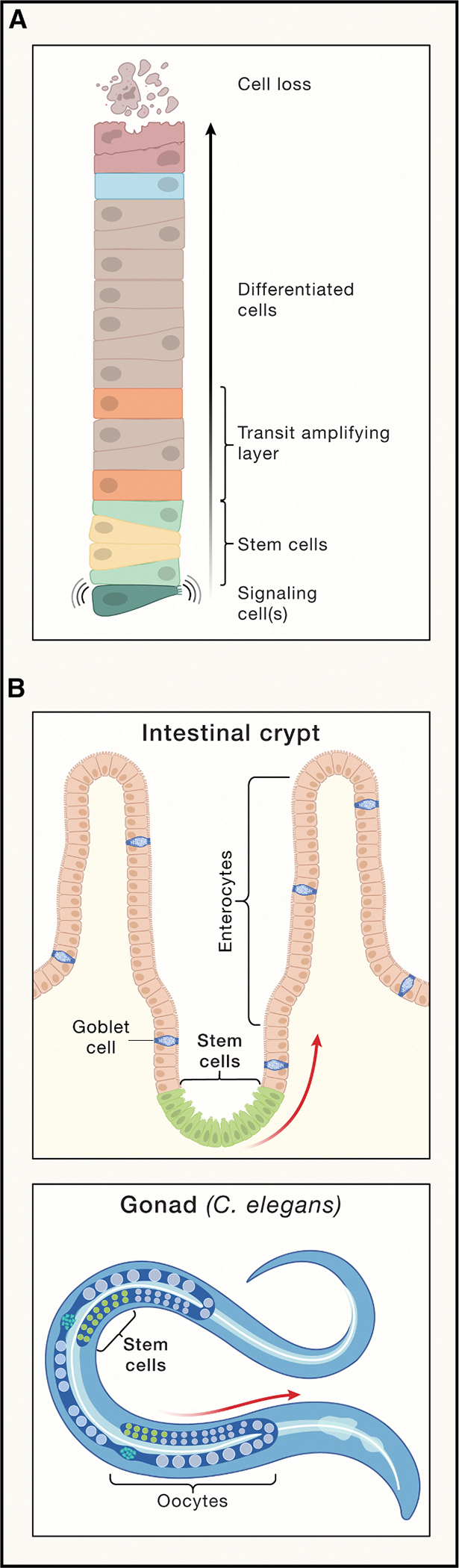
Systems for producing mature, functional cells from stem cells at a controllable rate They compensate for variable cell loss and maintain the appropriate numbers and types of cells in a tissue or organ.

At first glance, maintaining tissue homeostasis—balancing cell production and loss—appears straightforward. In practice, however, it requires remarkable cooperation among cells. Any set of dividing cells (e.g., stem cells) will produce a constant output of post-mitotic cells only if exactly half of their progeny differentiate and half do not. Deviations from this balance drive dividing cells either toward eventual extinction or unchecked, exponential expansion. Less widely appreciated is the fact that it is not good enough for cells to achieve this balance merely on average (sometimes differentiating too rarely, sometimes too often), because fluctuations introduced into an exponential process (like cell proliferation) necessarily compound over time, leading to an ever-growing variance in tissue size (Figure 3).^61^

This leaves evolution to seek out either of two solutions: either every dividing cell must always divide asymmetrically, producing exactly one differentiated cell each time, or dividing cells must cooperate with each other through some sort of feedback mechanism that counteracts random fluctuations. It used to be assumed that the first strategy was the more common—textbooks still commonly portray stem cells as obligate asymmetric dividers—but it has become increasingly clear that many stem cell systems behave probabilistically, implying the need for feedback control.^62^

Interestingly, in implementing either of these strategies, nature seems to have repeatedly used a common motif, which we call the “proliferative conveyor.” In it, cells are confined within a fixed geometry, typically a cylinder, with one sealed end and one open one. Stem cells always reside at the sealed end, or base, and a stream of differentiated cells emerges from the open one. Examples, which can be found in both developmental and physiological contexts, include intestinal crypts (producing intestinal epithelial cells),^63^ hair follicles (producing hair),^64^ the growth plates of long bones (producing chondrocytes),^65^ gonads (producing gametes),^65^ and the presomitic mesoderm of vertebrate embryos (producing somites).^66,67^

Although these systems vary in many details—whether the tube is filled or hollow, circular or flattened, containing just a single layer of cells or many—each seems designed to harness proliferation to produce a steady output at a controlled rate. Typically, a special non-dividing cell or group of cells is found at the base, which provides signals to stem cells. In some cases (e.g., the *Drosophila* gonad), this spatial asymmetry is leveraged to ensure perfect division asymmetry by every stem cell.^68,69^ By contrast, in cases in which stem cells divide stochastically (e.g., the intestinal crypt^70,71^ and the mammalian testis^72^), it is likely that such cells are involved in helping stem cells coordinate with each other.

An inevitable consequence of enforcing balanced divisions on stem cells is that progeny can only be produced at a rate of one differentiated cell per stem cell per cell cycle. In situations of high demand, this is unsuitable. Thus, proliferative conveyors often interpose a “transit amplifying layer,” a proliferative progenitor stage between stem cells and terminally differentiated cells. Of course, proliferative progenitors face the same controllability issues stem cells do: to produce a reliable output stream, each cell needs to amplify by a reliable amount. In many proliferative conveyors, such progenitors get long-range signals from the base. For example, in the *C. elegans* gonad, proliferating cells are contacted by dendrites from the distal tip cell, and as proliferation conveys them away from the base, they lose such contacts—and the Notch signal they transmit—and undergo terminal differentiation.^73^

There exist examples of proliferative stages that are organized into spatially distinct layers in structures other than quasi-cylindrical proliferative conveyors, e.g., the vertebrate epidermis and the subventricular zone of the brain. Feedback strategies have been described that ensure that stem cells stay locally balanced in such sheet-based tissues,^74^ but how such tissues achieve global balance (i.e., how they avoid growing or shrinking along their planar dimensions) is still a mystery. By contrast, proliferative conveyors are discrete structures, usually generated during development. As long as they are made in the right numbers and each produces a controlled output, global control is assured.

#### Repeaters

Tissues continually sense stimuli from their external environment, adapting their functions to shifting circumstances to promote host fitness. Achieving this objective involves multiple cell types mounting coordinated responses over time and space. However, tissues face several challenges in this regard. For example, stimuli can be distributed across large regions of space, diluting the amount of input that any individual cell receives. This dilution can compromise signal-to-noise ratios, making it difficult to achieve accurate decision-making at the single-cell level and, in turn, a coordinated response across large areas of tissue. A second challenge relates to the fact that stimuli themselves are not necessarily uniform: they can exhibit fine or coarse-grained features throughout space, requiring varying degrees of detection (spatial) resolution to ensure appropriate tissue responses. How do tissues maintain adequate sensitivity and resolution toward noisy signals that can vary in their spatial granularity?

**Figure 4.**
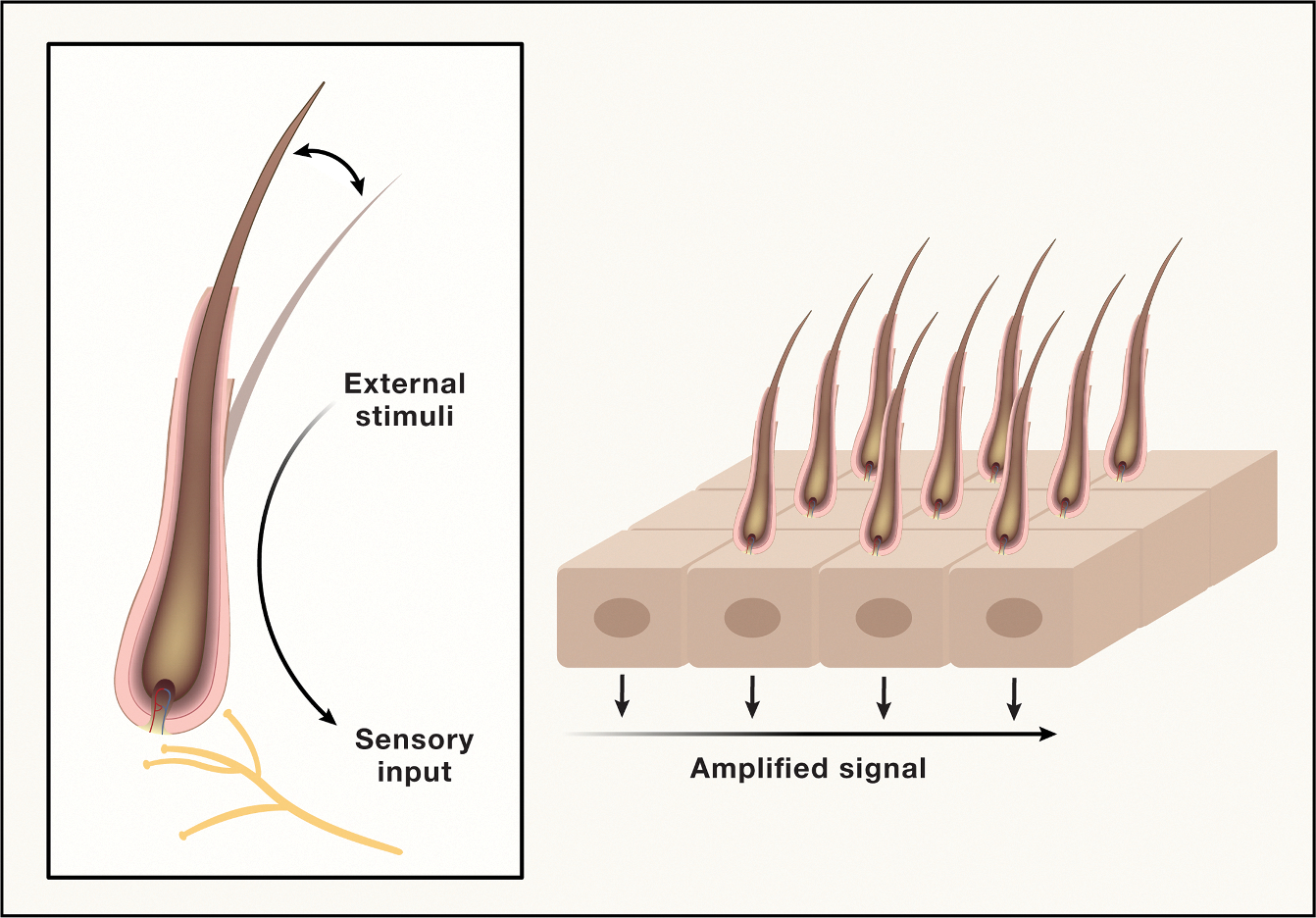
Uniformly arrayed biological structures distributed evenly over space Tissue organization emerges from the repetition of a minimal functional unit.

One strategy involves employing arrays of multicellular sensory units that repeat uniformly across space (Figure 4). This strategy has two advantages: (1) individual units can achieve higher detection sensitivity than their individual cell constituents in isolation,^75,76^ analogous to how receptor clustering can improve the sensitivity of signaling in individual cells,^77,78^ and (2) the joint evaluation of multiple sensory units in the array can improve detection resolution, enhancing a tissue’s ability to discriminate between different types of stimuli.^77–79^ Two classic examples of arrayed sensors are the mechanosensory and visual systems of insects.^80,81–83^ In these sensory systems, the single sensory unit (bristle or ommatidium, respectively) is repeated hundreds of times over in a highly ordered fashion. Notably, depending on the specific organism and tissue, the spatial frequency of individual units appears to depend on their sensitivity to stimuli, resulting in unique trade-offs. For example, a dense array of less sensitive units can be more robust to noise compared with sparse arrays of highly sensitive units.^80,81–83^

Apart from their role in improving surveillance of the external environment, repeating units help tissues overcome a range of additional challenges. For instance, because individual units can operate independently of one another, they can enable local tissue behaviors that are easier to control, compared with global behaviors, due to reduced length scales of activity.^79^ Local control is especially important for dealing with challenges like wound healing, where afflicted regions of tissue aim to reestablish their original spatial architecture. Consider the formation of pigment stripes in zebrafish—a classic example of a repeating pattern.^84^ Upon disruption of the stripe pattern by laser ablation, dynamic rearrangements in the remaining cells regenerate the stripe pattern according to local rules, consistent with the mathematics of reaction-diffusion-mediated self-organization.^85^ Similar results are observed for the regeneration of rugae stripes in mouse palates.^86^ Lastly, the presence of repeating functional units may facilitate effective monitoring of a tissue in its entirety, enabling accurate measurements and corresponding feedback control of organ size. A putative example can be seen in the liver, an organ comprised of repeating functional units known as portal lobules that are essential for the production of bile. The liver possesses a remarkable capacity to regenerate itself following partial hepatectomy, restoring 100% of its original size over the course of ~7 days.^87^ During this regenerative process, changes in the balance of bile acid reabsorption from the small intestine provide critical feedback control that stimulates liver growth.^88^ This behavior predicts that liver growth attenuates once the number of portal lobules reaches a threshold that supports sufficient bile production. These latter considerations overlap in part with the growth control issues raised in discussing proliferative conveyors.

“Repeaters” appear to solve multiple problems related to tissue surveillance, sensory discrimination, repair, and scaling. However, it is easy to envision how these higher-order functions could be co-opted or perturbed to promote disease. In this regard, recent studies suggest that pancreatic ductal adenocarcinoma (PDAC) cancers exhibit stereotypic patterning (e.g., pancreatic intraductal neoplasia) reminiscent of repeaters, with each unit comprising characteristic frequencies of distinct malignant cell states. Notably, these frequencies are maintained through feedback control. The net result is local cooperativity between the different cell states, driving a level of tumor growth and metastasis that cannot be achieved by any one state alone.^89^ There is evidence that repeating units of similar nature can be found in other cancers beyond PDAC, endowing solid tumors with various emergent properties, such as increased sensitivity to growth factors or enhanced immunosuppression.^90^ A better appreciation of these concepts should provide a helpful framework for understanding tumorigenesis and developing new therapies targeting the structure of individual units and the spatial organization of repeaters.

#### Search parties

**Figure 5.**
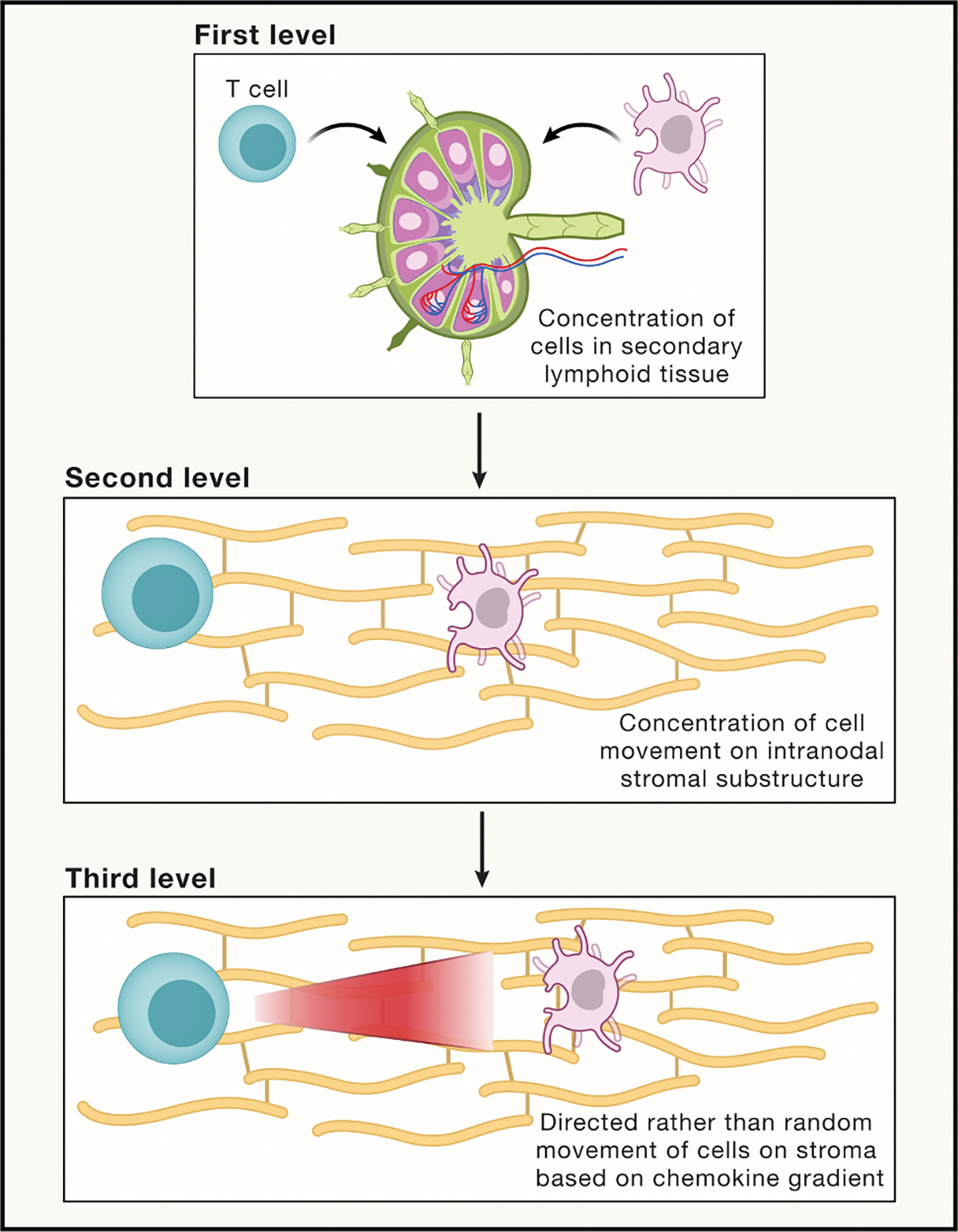
Coordinated movements of individual cells, often in substantial numbers, within or between tissues Search parties provide effector functions at a site of tissue damage or infection.

To promote host protection, immune cells migrate throughout tissues to search for foreign threats. While simple in concept, this process is a “needle in a haystack” problem given the unpredictable locations of infectious agents and their rarity in number, at least during the early stages of infection. Moreover, the size of a tissue usually far exceeds that of individual cells, leading to an enormous 3D search space.^91^ How do immune cells rapidly locate host threats? Unlike the majority of cells of solid tissues, which show limited 3D movement, most immune cells exhibit extensive motility in adult organisms, comparable to somatic cells during organogenesis in embryos. While such migratory properties can be beneficial, both computational and experimental studies have demonstrated that random motility of individual cells alone is an inefficient search strategy for locating rare threats.^92,93^ It is therefore unsurprising that dynamic imaging studies over the last two decades have revealed strategies involving the coordinated movements of multiple cells, herein referred to as “search parties.” These strategies often rely on positive feedback whereby activated immune cells release chemokines to recruit distant immune cells that, in turn, become activated and release even more chemokines—a process akin to how cyclic AMP (cAMP) organizes the migration of *Dictyostelium* cells in streams to form a fruiting body.^94,95^ A well-characterized example involves T cells during the earliest stages of an immune response in secondary lymphoid tissues. While searching for rare, unpredictable antigens presented by dispersed dendritic cells, T cells initially undertake random (or Levy) walks on the stromal networks (i.e., streets) previously mentioned.^96,97^ However, upon recognition of cognate antigen, individual T cells produce chemokines that attract other T cells, biasing their otherwise random walk to enhance interactions with relevant dendritic cells (Figure 5).^98–100^

While essential for ensuring rapid detection and elimination of rare targets, the inherent non-linearities of search parties pose a risk to the host. Indeed, concentrating activated immune cells in relatively small regions of space can enhance collateral tissue damage due to the focal release of inflammatory signals. This trade-off necessitates stringent control mechanisms to limit the initiation, duration, and amplitude of positive feedback. Although such mechanisms remain incompletely defined, they appear to operate both at the intracellular and, importantly, intercellular scales. A recently described example involves neutrophils, which initiate self-amplifying, highly directed swarming behavior upon sensing tissue damage or microbes.^101,102^ However, as these cells accumulate focally, they can inflict significant damage to the underlying tissue architecture. To limit this detrimental outcome during inappropriate contexts, immobile tissue-resident macrophages extend cellular processes to “cloak” small lesions that occur naturally in healthy tissues, preventing the initiation of neutrophil swarming. The efficacy of cloaking becomes limited only once lesions exceed a specific size or local density, permitting the inflammatory response of neutrophils to operate unimpeded.^103^

Not all cellular traffic starts out undirected. Many cell behaviors require migration of cells from one tissue site to a distant one. These movements take advantage of streets, including blood and lymphatic vessels, to move from one organ, such as lymph nodes or bone marrow, to another, particularly parenchymal organs, including the skin, liver, kidney, and so on.^104^ To enter the parenchyma of organs, circulating immune cells must exit the vasculature—a process that depends on the sensing of local tissue damage, cellular and ECM-associated adhesion molecules, and chemoattractants. Active modifications of the intercellular junctions in the vascular endothelium must also occur in concert.^104^ Upon successful transmigration, search parties can form rapidly within the peri-adventitial region of the vessels and subsequently migrate to a site of infection or tissue damage.^104^

The application of *in vivo* imaging methods such as two-photon microscopy, in concert with genetic engineering of cells and animals, has led to substantial knowledge about the molecular control of search parties in diverse tissue settings as well as some insight into how these behaviors support immune homeostasis and effector function. However, we still lack a framework that quantitatively explains the coordination and dynamics of search parties. How much chemokine produced by how many cells in what spatial distribution is needed for proper guidance of immune cells in different situations? How are soluble and solid-phase chemoattractant gradients controlled across space? What factors constrain cell motility, given that the structure of a tissue varies with respect to cell packing and ECM components? Lastly, how is the number of immune cells that arrive in a tissue and form search parties tuned to deal with evolving threat levels (e.g., the amount of a given pathogen over time)? These are just some of the outstanding questions that require answers, impeding our understanding of how infections resolve or tumors regress.

#### Flocks, streams, and swarms

**Figure 6.**
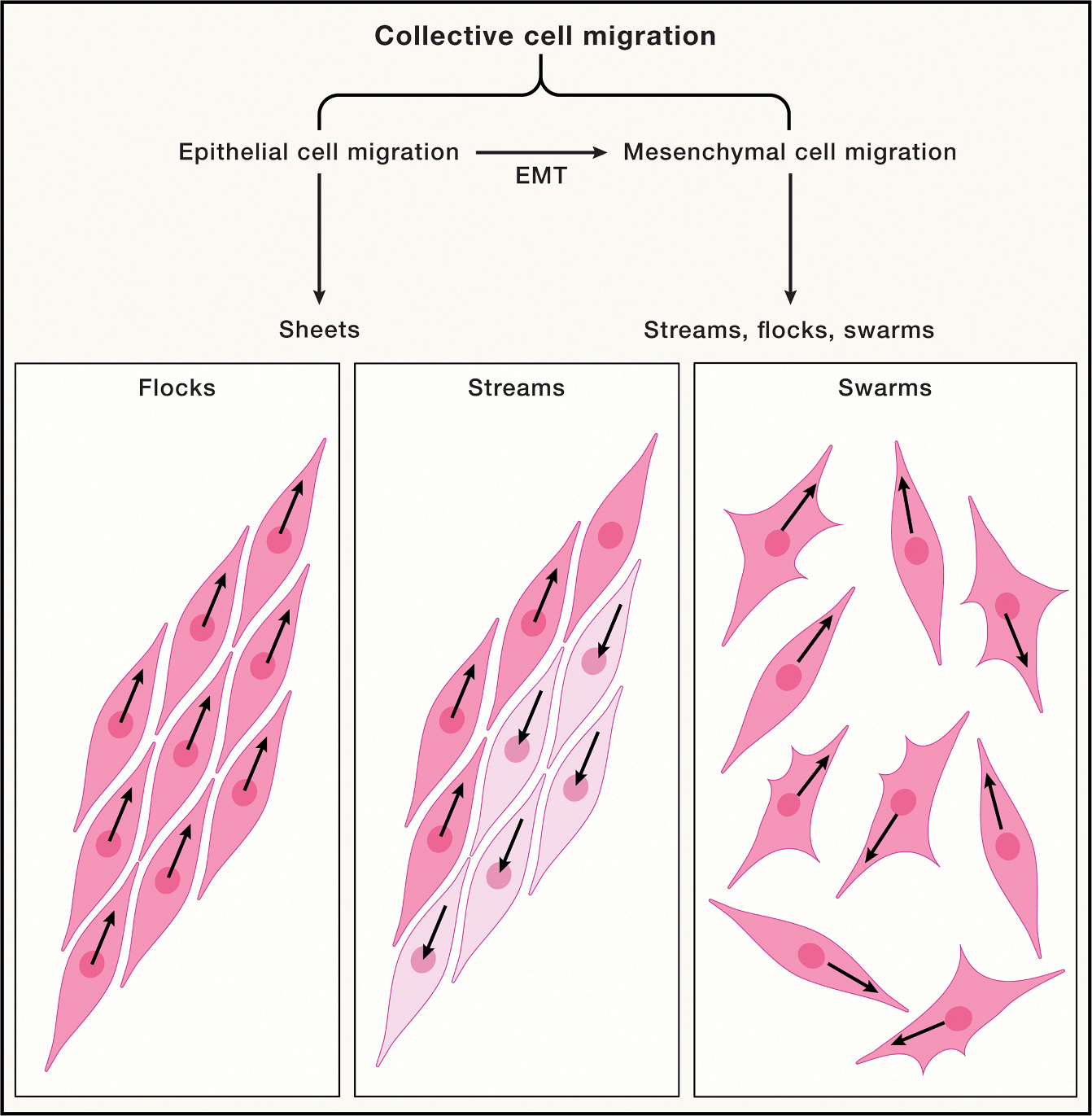
Units of collective migration exhibited by mesenchymal cells that facilitate tissue development and repair over large distances Such migration is frequently co-opted by cancer cells to facilitate tumor growth and metastasis.

While immune cells form search parties to pinpoint the location of threats, mesenchymal cells often employ different migratory strategies, adapted for distinct functional challenges, such as the orchestration of tissue remodeling during development and wound repair.^37^ These tasks involve long-range, coordinated movements of cells in a manner that ensures their seamless integration into evolving tissue architecture. One solution to this challenge is collective migration, which allows mesenchymal cells to move en masse, influenced by interactions with one another and the environment. This phenomenon manifests in diverse patterns reminiscent of “flocks, streams, and swarms”—characteristic types of motion that describe the behaviors of fluids, particles, and animals (Figure 6).^105–108^ For example, in vertebrate development, neural crest cells undergo a highly directional, stream-like migration following an epithelial-to-mesenchymal transition (EMT).^109,110^ This type of motion allows neural crest cells to reach specific regions of the embryo to form various structures, such as the peripheral nervous system and facial cartilage.^110^ Conversely, in wound healing, epithelial cells also undergo a transient EMT but then migrate to repair the wound with flock-like coordination, resembling birds in flight.^74,109^ This diversity in migratory behavior raises a pivotal question: what are the performance trade-offs of each migration pattern in different cell biological contexts?

Addressing this question is challenging, especially when much direct experimental evidence is lacking. Nevertheless, insights from physics, mathematical modeling, and ecology offer valuable, albeit speculative, frameworks. Flocking, characterized by its flexibility and coordination, mirrors how birds adapt to environmental changes.^107^ This trait likely aids mesenchymal cells during dynamic processes, such as wound healing, wherein a rapidly changing inflammatory landscape necessitates continuous adaptation. However, the inherent flexibility of flocking may not lend itself well to the formation of stereotyped tissue structures, such as those in neural or skeletal systems, which require more precise cellular arrangements. By contrast, stream-like migration offers a more structured, highly directional approach, resembling a river’s path.^111^ This mode could be advantageous for developmental processes requiring exact cell positioning, as seen with neural crest cells. Yet efficiency might come at the expense of adaptability, potentially limiting responsiveness to unexpected (and unpredictable) local environmental shifts. Swarms, by contrast, involve the local congregation of cells that actively migrate toward one another in response to environmental cues, forming dense, spatially organized clusters with minimal net displacement. This behavior is reminiscent of insect swarms, such as midges or honeybee clusters, which exhibit coordinated positioning but negligible collective displacement. While such organization limits long-range movement, it may enable extensive sampling and integration of local cues, thereby coordinating a focused tissue response.^111^ This strategy could prove valuable in collective sensing of spatial and temporal heterogeneity over long time and length scales, as is likely required in repair or regeneration of large, complex tissues such as the liver.

The inappropriate execution of flocking, streaming, or swarming migration likely plays an important role in tissue-scale diseases, especially cancer. For instance, in some colon and brain cancers, cancer cells of epithelial origin undergo EMT and acquire stream- and flock-like behaviors, which facilitate spread to distant organs and local invasion, enhancing metastatic potential.^37^ Such behavior may also enable cancer cells to remodel surrounding tissues to create conduits that facilitate further invasion or physical barriers that limit immune cell infiltration. This notion is supported by studies in genetically engineered models of glioma, where the prevalence of cancer cell streams within the tumor microenvironment has been linked to increased malignancy.^112^ Additionally, swarm-like movements observed within tumors may allow cancer cells to efficiently sense the ever-changing tumor microenvironment, optimizing their adaptations to facilitate growth and metastasis.

While the conceptual frameworks of flocks, streams, and swarms offer intriguing insights into the collective migration of mesenchymal cells, the performance trade-offs of each will require experimental investigation. Critical questions also remain about the mechanisms that sustain these migratory patterns, including key aspects of cell-cell communication, the frequency and nature of intercellular contacts, and the role of paracrine signaling. Unraveling these mechanisms is crucial for advancing biological knowledge that could facilitate the development of innovative therapies. For example, precision targeting of aberrant migration patterns in cancer could provide new strategies to curb metastasis, whereas harnessing beneficial migratory patterns could enhance tissue repair and regeneration following injury.

#### Spatial A-D converters

One view of tissue functions suggests they emerge from the actions of discrete cell types, with sharp boundaries between adjacent cell types,^113,114^ as opposed to a smooth continuum of cell states. Yet the signals that organize tissues in space are often continuous—for example, molecular gradients produced by morphogens and cytokines, which diffuse throughout 3D space to form concentration gradients that decay with various length scales.^115,116^ Turning graded (i.e., analog) information from signal-producing cells into sharply bounded (i.e., digital) local domains of cellular behavior implies that signal-receiving cells employ some form of analog-to-digital (A-D) conversion.^117–121^ The variegated division of labor that results from such conversion promotes many functions, including tissue development, metabolism, and effective host defense by the immune system.^117–121^

**Figure 7.**
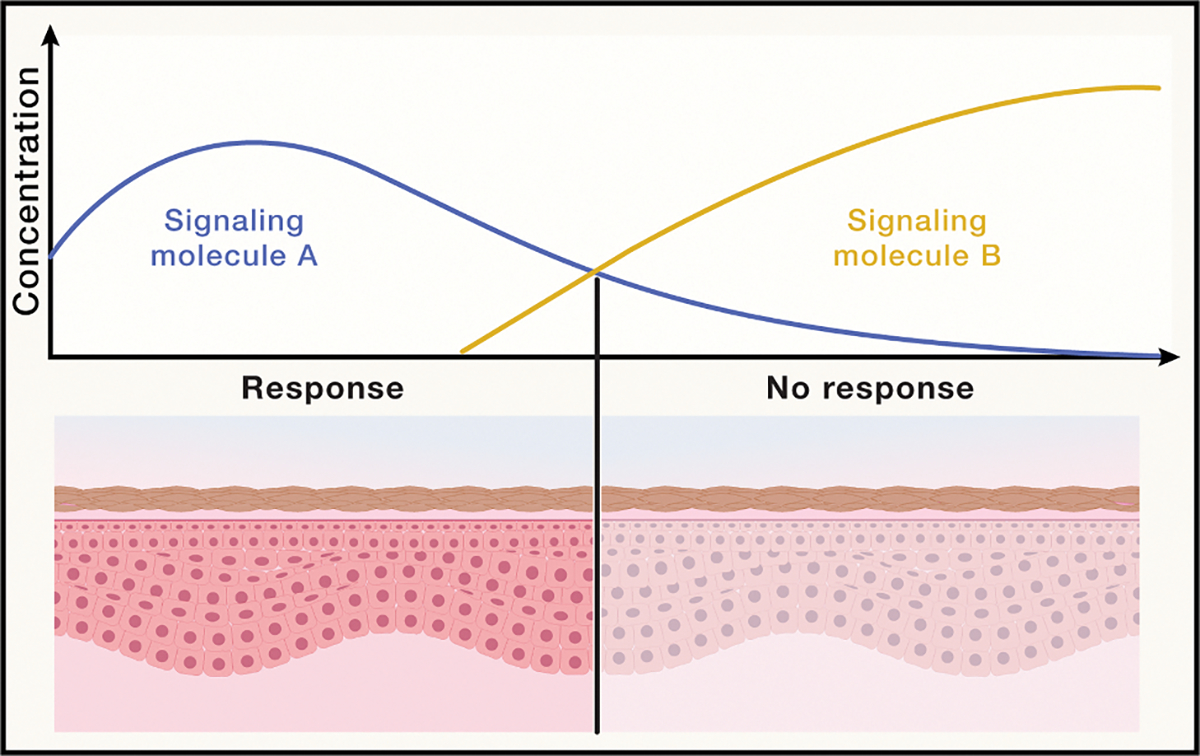
Multicellular units that convert graded, noisy input signals into discrete output responses at fixed coordinates in space

Ensuring the right group of cells achieves the correct A-D conversions at the correct spatial coordinates is rife with challenges, especially in the face of biological noise, which includes fluctuations within cells over time and variation between cells throughout space. These compounded intracellular and intercellular effects degrade the spatial precision and accuracy of A-D conversions.^116^ For instance, intracellular noise can cause seemingly identical producer cells to vary in their ability to synthesize or secrete diffusible signals and seemingly identical receiver cells to vary in their detection machinery.^74,122,123^ Intercellular noise also emerges due to stochastic fluctuations between surface receptors on receiver cells and their cognate diffusing ligands. Importantly, fluctuations in receptor occupancy increase non-linearly as concentrations of paracrine signals decline with distance from producer cells.^74^ In other words, noise is not uniform throughout tissue space. “Spatial A-D converters” are thus mesoscale modules that facilitate accurate A-D conversions in the face of spatially varying noise (Figure 7).

Spatial A-D converters appear to use a range of control strategies.^124^ For instance, feedback mechanisms operating between producers and receivers can preserve the spatial profile of a morphogen or cytokine gradient, despite cell-to-cell variation in producer cells, thereby stabilizing the coordinates at which specific A-D conversions occur. A classic example involves receiver cells increasingly removing a diffusing molecule from the extracellular environment in response to signaling induced by the molecule itself—an inherently non-linear process. As a result, receiver cells rapidly clear the diffusible molecule closest to the source of production, promoting robustness in the overall shape of the gradient.^119,125^ Receiver cells can additionally buffer noise in their ligand detection machinery by integrating multiple measurements across time and, more importantly, space itself. One form of spatial integration involves receiver cells secreting a secondary diffusible molecule in response to a primary diffusible molecule. Because this secondary molecule acts over shorter length scales, it can smooth out local fluctuations in signaling induced by the much noisier primary molecule, improving the accuracy of A-D conversions in groups of receiver cells with similar spatial coordinates.^116^ The best characterized example of this process occurs in the BicoidHunchback system, which controls patterning along the anterior-posterior axis of the *Drosophila melanogaster* embryo.^126–128^ It is tempting to speculate that immune cells also employ spatial integration strategies to control the location of A-D conversions given that many primary cytokines induce the production of secondary cytokines.^129,130^ Lastly, self-organization strategies, such as those arising from specialized reaction-diffusion models (e.g., Turing patterns), provide an elegant solution to spatially varying noise and have been documented in an increasing number of biological contexts.^84,116^

Ultimately, the strategies used by spatial A-D converters likely are optimized for distinct performance objectives.^74,115,131^ For instance, the location of specific A-D conversions in tissue patterning systems requires precision, accuracy, and robustness toward noise, presumably due to the fitness costs associated with developmental errors. By comparison, the location of A-D conversions during immune responses requires robustness with respect to variation in some parameters but significant plasticity (i.e., tunability) with respect to variation in others.^119,132,133^ These differences likely stem from the fact that the immune system must contend with pathogens that are diverse, dynamic, and unpredictable in nature. Looking forward, a systematic understanding of spatial A-D converters in distinct biological contexts—including development, cancer, infectious disease, and wound healing—will be required, with an emphasis on quantifying performance trade-offs. Such analysis will be enabled by recent advances in tissue microscopy, coupled with computational and experimental manipulations that can fine-tune key parameter values. In turn, accompanying changes in spatial gradients and the locations of specific A-D conversions can be quantified *in situ*. We expect this approach to yield insights into the emergence of disease states, particularly solid tumors, which frequently disrupt the locations of A-D conversions and, in turn, promote unnatural spatial patterning of cell states that perturb tissue physiology and immune functions.

### THE BENEFITS OF MODULARITY

The sections above outline the concept of mesoscale modules—multicellular units that are more complex than individual cells but simpler than tissues and organs—that fulfill distinct functions through specific structural and physiological arrangements (Table 1). Each module is defined by interactions among a modest number of cells and cell types, often embedded in or interfaced with specific extracellular matrices. For many modules, function is closely tied to specific spatial arrangements—how cells and extracellular components are positioned in relation to one another—which, in turn, constrain cell movement and interactions. These arrangements also shape how signals pass between cells, as the transport of molecules and forces over tissue-scale distances can operate in ways distinct from the cellular scale.

To illustrate how mesoscale modules are defined based on physiological functions and recur across tissues, we highlight the digestive tract as an archetypal example. Here, multiple module types—including sheets, streets, proliferative conveyors, repeaters, search parties, and spatial AD converters—work in concert to maintain gut homeostasis (Table 2). Notably, this “top-down” approach groups functionally similar structures into common categories, even when their cellular composition or developmental origin differs. For example, one-dimensional structures composed of ECM fibrils or elongated, interconnected cells are both categorized as streets, while epithelium and endothelium are both considered sheets.

Advancing our understanding of tissue spatial dynamics will undoubtedly uncover new modules beyond those discussed here, including structures with novel geometries, signaling logic, or mechanical functions. Identifying and characterizing such modules will require a combination of experimental innovation, computational modeling, and conceptual clarity. In the paragraphs that follow, we consider potential research directions, address key analytical and methodological challenges, and explore the broader implications of mesoscale modules for understanding complex tissue phenomena in both health and disease settings.

Because each module is defined through structure-function relationships, these frameworks may provide a practical toolkit for identifying the minimal design principles required for higher-order tissue functions. This perspective may also open new avenues for characterizing diseases in terms of their impact on common modules—for example, how tissue repair and oncogenic transformation influence conveyors, or how autoimmunity and tumor immune evasion affect search parties. By analyzing complex diseases like cancer, diabetes, and neurodegeneration through the lens of mesoscale modules, we expect to uncover functional abnormalities that are more directly translatable to diagnosis or treatment than molecular markers alone.

Our focus on mesoscale modules is motivated, in part, by the conviction that biological systems are organized hierarchically, with these modules serving as foundational subunits for the emergence of higher-order functions. Accurately predicting these functions will require characterizing mesoscale modules with the same degree of mathematical precision that systems biologists have applied to intracellular network motifs. Achieving this precision will, in some cases, demand a deeper understanding of the molecular signals underlying processes such as tissue mechanics and cell polarity. In other instances, existing supracellular theories, such as Turing patterning, may provide a strong starting point for exploring self-organization.^142^ These efforts hold the promise of greatly enhancing our ability to model and manipulate tissue-level behaviors in both health and disease settings.

Enumerating mesoscale modules is not merely a way to categorize tissue behaviors but a framework to guide future research. Lessons from the study of intracellular processes support the utility of modular thinking. For example, the expectation that complex cellular behaviors could emerge from mathematically tractable network motifs spurred discoveries of patterns such as thresholds, oscillators, and adaptation.^143,144^ A modular approach also drove critical innovations in data acquisition and analysis, including the transition from static to dynamic measurements.^145^

In an analogous way, a focus on modularity at the supracellular scale could catalyze breakthroughs in understanding tissue-level organization and behavior. For instance, an awareness of mesoscale modules could help researchers anticipate patterns in single-cell transcriptomics, genomics, and proteomics, particularly as spatial “omics” methods become more prevalent. Consider the concept of “niche,” which has historical origins in models of stem cell function but has become increasingly defined in the practice of spatial omics as small neighborhoods composed of recurrent combinations of cell types defined by gene expression profiles.^146–158^ While undoubtedly useful, there is no guarantee that such spatial patterns of gene expression map neatly onto units of tissue-scale function. By contrast, the mesoscale modules proposed here are defined by function first—barrier formation, cell trafficking, signal detection, tissue growth—with corresponding descriptions of minimal structural and cellular requirements to achieve them. Such modules can include more than just cells (e.g., ECM), are defined by physical features beyond mere proximity (e.g., detailed geometry and physical constraints), and emphasize the behaviors that emerge from cell collectives, not just those that emerge from single-cell molecular states.

Interpreting single-cell data through the lens of mesoscale modules shifts the analytical paradigm from purely unsupervised discovery to a more supervised, hypothesis-guided approach. At first glance, this shift may appear paradoxical—why would gathering more diverse and detailed data require supervised approaches to uncover biologically meaningful insights? The answer lies in the additional dimensions introduced by spatial features, specifically the three coordinates of physical space, which dramatically expand the set of biologically plausible configurations. While clustering algorithms can effectively group cells by transcriptomic similarity, spatial data demand consideration of intercellular relationships, positional context, and molecular gradients—factors that significantly expand the complexity of analysis. Should we account for the distance between every pair of cells? What about the combinations of cells interspersed between others, their orientation or asymmetry, or their periodicity at particular spatial scales? The relationships of cells to structural cues, such as blood vessels, lymphatics, nerves, or the ECM? Or the spatial distributions of cell surface receptors that influence signaling gradients? Even in a small piece of tissue, the sheer number of possible spatial relationships between cells is daunting, making it impractical to test for the co-dependence of everything, everywhere, all at once.

These considerations highlight how the leap from pixels and cells to multicellular units is much larger than it may seem, requiring conceptual and analytical frameworks beyond those used to assess single-cell gene expression variation. While promising efforts to address these challenges are emerging, they remain in their infancy.^137,159–162^ For instance, methods that focus on identifying recurring combinations of cells within small, regular areas or “neighborhoods”^137,162^ are typically limited to detecting two-dimensional patterns, likely failing to capture the full complexity of 3D tissue structures. Moreover, methods designed to detect spatial structure in static images may fail to capture dynamic phenomena—such as signaling or mechanical feedback loops—that emerge from cell-cell cooperation or cell-matrix interactions.

In principle, existing unsupervised methods, including graph-based and neighborhood-clustering tools, could be extended to account for additional features such as tissue geometry, cell polarity, ECM composition, or multicellular junctions—factors that may be critical for identifying functionally coherent units.^146–158^ However, in the absence of labeled training data, even these more sophisticated methods may struggle to detect complex, spatially organized modules without access to rich, multimodal datasets—particularly those capturing 3D structure or extracellular context. In addition, the absence of biological priors may make it difficult for unsupervised approaches to infer functionally meaningful units (i.e., those with emergent behaviors) from spatial data alone. Recent information theory-based methods offer a promising, more flexible form of unsupervised discovery, though their utility in detecting functionally defined modules remains to be fully tested.^159^

A similar trade-off between supervised and unsupervised approaches also existed in the context of module discovery at the cellular scale. For example, the network motifs identified by Alon^28^ and Davidson^163^ were initially derived through graph-theoretic, bottom-up analyses of interaction networks. Yet the enduring value of these motifs stemmed from the functional behaviors they exhibited—such as feedback, oscillation, or robustness—not from structure alone. Analogously, bottom-up computational approaches may eventually prove essential for defining mesoscale modules, but doing so will require adapting existing tools to accommodate the vastly greater complexity of tissue-scale systems. Meeting this challenge will likely demand concerted efforts from the systems biology community to develop new theoretical frameworks and analytical pipelines that scale with the number of nodes, spatial dimensions, and combinatorial features involved.

An intermediate path may therefore lie in supervised analyses, where computers are biased toward recognizing biologically meaningful patterns—including cell locations and gene expression as a function of spatial context.^164^ Mesoscale modules, with their manageable size and defined structure-function relationships, are ideally suited to bridge this gap. Their intermediate scale may provide the right level of complexity to exhibit emergent behaviors without being overwhelmed by combinatorial possibilities. Still, a critical open question is whether such approaches can be feasibly implemented without relying on extensive and time-consuming manual annotation of training data.

Overall, we hope this article has succeeded in highlighting the value of studying tissue-level behaviors from the perspective of mesoscale modules. Our exploration here is, of course, not exhaustive. While we focused on spatial patterns, it is equally important to recognize that temporal patterns also emerge at the tissue level. Well-known examples include peristalsis in the gastrointestinal tract and gamma waves in the brain. Peristalsis physically moves ingested food through the digestive system via a coordinated temporal sequence of actions involving smooth muscle cells, interstitial cells of Cajal, the enteric nervous system, enteroendocrine cells, and epithelial cells.^165^ Gamma waves, on the other hand, are associated with critical brain functions, including cognitive processing (e.g., problem solving), memory formation, sensory perception, and motor control. These waves arise from synchronized neural oscillations when groups of neurons fire at frequencies between 30 and 100 Hz. This synchronization, mediated by GABAergic interneurons, depends on the precise timing of collective neural firing.^166^ Notably, the emergence of tissue-level temporal patterns depends on appropriate spatial organization to facilitate intercellular coordination. It is therefore unsurprising that disruptions in spatial organization are often linked to diseases, such as irritable bowel syndrome (IBS)^167,168^ and epilepsy,^169^ which are associated with abnormal peristalsis and gamma waves, respectively. While the roles of emergent temporal patterns warrant deeper exploration, they are beyond the scope of this perspective.

Future biologists may look back on the 20^th^ century as the era of the cell, when the fundamental principles of cellular function first came into sharp focus. Now, in the 21^st^ century, the opportunity to apply a similar degree of scrutiny to tissues has arrived. As in every era, scientific progress depends on developing tools for observing and measuring processes at the appropriate scale and creating useful conceptual frameworks to interpret the resulting data. In recent years, single-cell biologists have made spectacular advances in achieving the former. Characterizing tissue functions in terms of properties that emerge from simple, mesoscale modules should help to achieve the latter.

### CONCLUDING REMARKS

Understanding how tissue functions emerge from the collective behavior of cells remains one of the central challenges in biology. Here, we propose that identifying and studying mesoscale modules—functionally coherent, multicellular units—can provide an essential bridge between modern single-cell data and tissue-level phenomena. We believe that conceptualizing tissues in terms of modular, emergent behaviors will offer a tractable path forward, particularly as spatially resolved transcriptomic and proteomic data continue to grow. However, realizing this vision will require the development of new analytical approaches that extend beyond current neighborhood-clustering methods, incorporating physical features such as geometry, polarity, and ECM organization. Defining mesoscale modules not only offers a framework for understanding healthy tissue organization but may also reveal how modular dysfunction drives disease. Ultimately, we hope that formalizing the mesoscale will enable a more predictive and mechanistic understanding of how cells collectively create robust, adaptable tissues. The critical challenge ahead is to explain how higher-order tissue functions emerge from the integration of molecular, spatial, and mechanical information across scales.

## Figures and Tables

**Table 1.** Proposed mesoscale modules

Mesoscale module	Functional task	Parts list	Parameters	Examples	Constraints	Microscale analogs

**Sheets**	provide selective barriers, facilitate long-range mechanosensing	cells, cell-cell junctions, ECM	spatial scale, cell polarity, strength of junctions, adhesion types, basal lamina structure and mechanical properties, cell mechano-sensing pathways	endothelium, mesothelium, surface epithelium	enforced mechanical coupling, enforced cell polarity, constrained motility, acute need for size control	ion channels (on a membranous organelle)
**Streets**	expedite long-range transport of cells and macromolecules	ECM fibrils, interconnected cells	fibrous architecture, diffusivity, gradient (for chemotaxis, durotaxis, haptotaxis, and curvotaxis)	elongated stroma in lymph nodes, lymphatic and blood vessels, axon bundles	ECM fiber density, pore size, orientation, and proteoglycan content	intracellular transport along microtubules
**Conveyors**	produce a consistent population of differentiated cells and robustly maintain tissue/ organ size	stem cells, progenitor cells, differentiated cells, intercellular signals	cell division rates, renewal probabilities, renewal asymmetry, clone sizes, cell polarity, asymmetry of arrangement	intestinal crypts, hair follicles, testis, epidermis, long bones, developing somites	niche dimensions, structural constraints, stochastic extinction, limits to the ranges of intercellular signals	coordinating production of ribosomes with production of structural proteins
**Repeaters**	extend sensory detection over large spaces, enhance resolution of detection, filter detection noise, measure tissue size	multi-cell functional units	transport of diffusible signals, degradation kinetics, target gene expression, differences in receptor expression or cell protrusions	gastrula, palate, bristles, hairs, alveoli, hepatic triads, lobules, cortical sulci, distribution of astrocytes and microglia throughout the brain	niche dimensions, structural constraints	clusters of receptors on the cell surface
**Search parties**	samples the local environment to quantify the density of specific biomolecules or cell types	symmetry breaking through cell-cell interactions	chemoattractant signals, affinities, cell motility	lymphoid cells, myeloid cells, regulatory T cells	availability of streets	multiprotein signaling hubs concentrated on the intracellular side of the plasma membrane
**Flocks, streams, and swarms**	allow collective movement of cells in either one or two directions	constituent cells, cell-cell adhesions	strength of cell-cell attachments, number and identity of leader cells, migratory velocity, cell polarization	wound healing, immune responses, embryogenesis, migration of neural crest cells, trachea branching, tumors	dimensions of migratory channels (e.g., neural tube), cell shape, cell density	networks of microtubules that facilitate cytoplasmic streaming
**Spatial A/D converters**	create sharp boundaries and discrete cell types from smooth, analog signaling gradients	diffusible signals, feedback loops, distinct dynamic attractors, biological noise at large scales, detector cell sensitivity	cell states, position, gradients, intrinsic length scales	morphogen gradient systems, left-right pattern, immune/autoimmune boundary, Turing patterns	signal-to-noise issues, speed vs. accuracy trade-offs, fitness costs of developmental errors	bistability in cell states generated through positive feedback and hysteresis

ECM, extracellular matrix.

**Table 2.** Modules in the digestive system for homeostasis maintenance

Module	Example in the GI tract	Function

Sheet	mucous epithelium	the epithelium serves as a physical barrier against the external threats—including pathogens and irritants—while also buffering mechanical impacts^134^
Street	Peyer’s patches	in the Peyer’s patches, stromal cells form chemokine-rich reticular networks that guide T cell migration via CCR7 signaling^135^; these networks likely function as streets—physically and chemically directing immune cell movement to facilitate antigen surveillance— similar to those in other secondary lymphoid tissues^56^
Proliferative conveyor	intestinal crypt	the stem cells continuously divide to generate enterocytes, goblet cells, and enteroendocrine cells, all of which migrate along the crypt- villus axis, replacing older cells shed from the villus surface^136^
Repeater	intestinal villus	the repeating villus units are critical for maximizing surface area and ensuring efficient nutrient absorption; this highly organized pattern not only optimizes physical contact with luminal contents but also enhances the tissue’s sensitivity to dietary changes; upon shifts in nutrient availability, villi across the intestine uniformly reconfigure their epithelial cell-type zonation along the crypt-villus axis, enabling coordinated adaptation across the tissue^137^
Search party	neutrophil swarms	following Salmonella infection, neutrophils rapidly migrate into the gut lumen and form dense clusters^138^; these clusters are reminiscent of feedback-driven neutrophil swarms observed in other tissues during infection or injury^102,139^
Spatial A/D converter	crypt-villus axis patterning via Wnt	in the intestinal epithelium, a graded and inherently noisy Wnt signaling gradient specifies discrete cell fates along the crypt-villus axis; multiple regulatory mechanisms—including spatially organized sources of Wnt ligands and antagonists as well as feedback loops involving Notch signaling—appear to buffer this noise to ensure robust A-D conversion of positional information into distinct cellular identities^140,141^
